# Practical Notes on Diagnosis and Treatment

**Published:** 1910-10-01

**Authors:** 


### Practical Notes on Diagnosis and Treatment.

**Water-brash.**

A regurgitation of acrid fluid into the mouth is practically pathognomonic of simple trouble; it is very rarely indeed seen in organic disease.—*Dr. Montague Murray.*

**Angina in Gouty Patients.**

Angina in the gouty requires our most penetrating attention, and there are those who require, even, it may be, in excess of their usual habit, perhaps one of those very things that in the unwisdom of routine we might be inclined to cut off. Certainly there are some to whom port wine, champagne, or cream or butter or sweetstuff will, each in its place and opportunely, bring healing on its wings.—*Dr. Goodhart.*

**Acute Polyarticular Rheumatoid Arthritis.**

The acute type usually occurs in children or young adults, and the chief changes are in the synovial membrane of the joints. The attack usually commences with pain and swelling and some rise of temperature. The smaller joints are those most frequently affected. Often a slow progressive swelling commences in the metacarpo-phalangeal joints of the index and middle fingers, and it may spread from these to others until most or all joints are involved. The pain is often persistent and tends to be increased at night. The attack may follow on one of acute rheumatism.

**Treatment of Pneumonia.**

The great needs of the body in pneumonia are plenty of air, water, and rest. Over-feeding and wrong feeding are responsible for a loss of energy used up in an attempt to digest, assimilate, and excrete unsuitable foods. Meat broths are not useful because they make no energy and tax the kidneys. Sugar is a valuable energy-producing food and leaves nothing but water and carbon dioxide to be eliminated. Failure to keep the patient in a horizontal position so as to aid the heart in carrying on the circulation is responsible for many deaths.—*Dr. G. Werley.*

**The Skin Diseases of Gout.**

These fall into two main groups. Those associated with the early periods in which the errors of metabolism are most in evidence, and those which occur in association with the period of arterio-sclerotic change. In the earlier stages there occurs a pruriginous scaliness of the skin which shows itself mainly on the extremities; this becomes a dermatitis, and eventually a true eczema of the gouty is established. In the more advanced stages of gout the more common manifestations are slight pityriasis and general exfoliative dermatitis. The chronic eczema of the gouty must be watched to avoid the risk of its degenerating into secondary exfoliative dermatitis. As the result of vascular changes, eruptions of purpuric character are not uncommon, and indicate a grave prognosis.—*Dr. James Galloway.*

**Digitalis.**

It is to be hoped that in time it will be generally regarded as very rash to give digitalis without taking the blood-pressure. In certain cases digitalis may cause deplorable results owing to its vaso-constrictor effect, as in the case of a heart that is failing behind a pressure which is already excessive. In such a case a nitrite must also be given to act as a vaso-dilator.—*Dr. Langdon Brown.*

**Digestion in Babies.**

I wish to protest against the view that babies cannot digest a small proportion of carbohydrate material, and the absurd fuss made by scientific baby-feeders against such an addition. These very gentlemen themselves have to recommend the addition of barley-water to cow's milk, and this contains more, and worse prepared, starch than do some of the best proprietary foods.—*Dr. F. J. Smith.*

**Pneumonia.**

If in a case of pneumonia the temperature fails to drop to the normal within eight days, we should remember the possibility of acute pneumonic tuberculosis; whereas if the temperature drop somewhat at the end of six or seven days and then takes a higher level, whilst the daily oscillation becomes greater, we should make an anxious search for the presence of an empyema.—*Dr. Arthur Latham.*

**Oxaluria.**

This by no means necessarily requires any treatment at all. The chief circumstances under which active measures may be required will be when there are signs of irritation of the urinary passages, in some cases of nocturnal enuresis, and in cases of mulberry calculus. It is necessary (*a*) to ensure the urine is not too concentrated by ordering plenty of fluids to be drunk, (*b*) to prevent undue acidity of the urine by giving bicarbonate of soda, (*c*) to keep up the proportion of magnesium in the urine by giving magnesium sulphate in small doses, (*d*) to avoid spinach, rhubarb, too much tea, and hard drinking water.

**Intravenous Injections of Adrenalin in Heart Failure.**

The influence of intravenous injections of adrenalin in cases of heart failure and of shock have been investigated by more than one observer, and the general conclusion come to is that the injection may restore life in a patient who seems to be moribund, and this even after other therapeutic measures have failed. The importance of this in a case of poisoning by anæsthetics and so forth is obvious, and the same applies to certain cases of pneumonia, heart failure, typhoid fever, acute poisonings, and so on. The surface of the mucous membranes become blanched, the blood-pressure rises, the pulse becomes stronger and slower, and the improvement may last from two to three hours.

